# Factors Associated with the Use of Different Treatment Modalities among Patients with Upper Airway Diseases in Taiwan: A Cross-Sectional Survey Study

**DOI:** 10.1155/2013/720879

**Published:** 2013-09-08

**Authors:** Malcolm Koo, Kai-Li Liang, Hsin Tsao, Ting-Ting Yen, Rong-San Jiang, Yueh-Chiao Yeh

**Affiliations:** ^1^Department of Medical Research, Buddhist Dalin Tzu Chi Hospital, No. 2, Minsheng Road, Dalin, Chiayi 62247, Taiwan; ^2^Dalla Lana School of Public Health, University of Toronto, No. 155, College Street, Toronto, ON, Canada M5T 3M7; ^3^Department of Otolaryngology, Taichung Veterans General Hospital, No. 1650, Sec. 4, Taiwan Boulevard, Taichung City 40705, Taiwan; ^4^School of Medicine, Chung Shan Medical University, No. 110, Sec. 1, Jianguo North Road, Taichung City 40201, Taiwan; ^5^Department of Medicine, National Yang-Ming Medical University, No. 155, Sec. 2, Linong Street, Taipei 11221, Taiwan; ^6^Graduate Institute of Natural Healing Sciences, Nanhua University, No. 55, Sec. 1, Nanhua Road, Dalin, Chiayi 62249, Taiwan; ^7^Department of Natural Biotechnology, Nanhua University, No. 55, Sec. 1, Nanhua Road, Dalin, Chiayi 62249, Taiwan

## Abstract

Rhinitis is a common upper airway disease and can have great impact on patients' quality of life. Factors associated with the use of common treatment modalities among 279 Taiwanese rhinitis patients from the outpatient department of otolaryngology in a medical center were investigated using a cross-sectional survey study. Results from multiple logistic regression analysis, adjusted for etiologies of rhinitis, revealed that males were associated with surgical intervention (OR = 2.11, *P* = 0.009). Lower educational level was associated with oral (OR = 2.31, *P* = 0.024) and topical medications (OR = 2.50, *P* = 0.005). Poor or fair general health status was associated with topical medications (OR = 4.47, *P* = 0.001), whereas very good or excellent general health status was inversely associated with surgical intervention (OR = 0.32, *P* = 0.002). Smoking was associated with the use of nasal irrigation (OR = 2.72, *P* = 0.003). Worse disease-specific quality of life was associated with oral medications (OR = 2.46, *P* = 0.010) and traditional Chinese medicine (OR = 5.43, *P* < 0.001). In conclusion, the use of different treatment modalities for rhinitis was associated with different combinations of independent factors.

## 1. Introduction

Rhinitis is the inflammation of the mucous lining of the nose, and it can lead to symptoms including rhinorrhea, nasal obstruction, post-nasal drip, itching, and sneezing. The etiologies of rhinitis include infection, anatomical anomalies, immunological disorders, hormonal imbalance, and ciliary defects [1, 2]. Similar nasal symptoms can be caused by different etiologies, making the diagnosis and treatment of rhinitis difficult. 

Methods for management of rhinitis include environmental control, pharmacotherapy, immunotherapy, surgical interventions, nasal irrigation, complementary, and alternative medicine [3]. Medications used for rhinitis are usually administered intranasally or orally. The surgical indications for rhinitis include drug-resistant inflammatory mucosal hypertrophy, anatomical anomalies, and sinus drainage obstruction. Complementary or alternative medicines such as traditional Chinese medicine (TCM), acupuncture, herbs, and probiotics are also being used for the treatment of rhinitis [4–6]. Recently, evidence-based guidelines with several algorithm-guided therapeutic schemes for the treatment of rhinitis are available [2, 7, 8]. Yet, to our knowledge, no studies have been conducted to determine the factors associated with the use of different treatment modalities. Therefore, the aim of this study was to investigate the factors associated with the use of treatment among patients with rhinitis in Taiwan.

## 2. Methods

### 2.1. Study Design and Subjects

Patients with physician-diagnosed rhinitis from the outpatient department of otolaryngology in Taichung Veterans General Hospital, a medical center in central Taiwan, were invited to participate in this cross-sectional survey study. The diagnosis of rhinitis was based on patients' reports of typical nasal symptoms persisting for two weeks or more and rhinoscopy examination. Typical nasal symptoms include rhinorrhea, nasal obstruction, postnasal dripping, itching, and sneezing. Physical examination with anterior rhinoscopy or nasal endoscopy was performed by two rhinologists (RSJ and KLL). All enrolled patients revealed signs of nasal inflammation including mucosal edema, nasal polyp, polypoid swelling, discharge (purulent, mucous or serous), or crust. Patients under 20 years old or with sinonasal tumors were excluded from the study. The study was approved by the Institutional Review Board of Taichung Veterans General Hospital, and all participants gave written informed consent.

### 2.2. Data Collection

Each patient completed a questionnaire with questions on sociodemographic status, lifestyle, general health status, disease-specific quality of life, and previous use of treatment modalities for rhinitis. The treatment modalities were classified into four main categories: pharmacology, surgical intervention, TCM, and nasal irrigation. Pharmacology was further subdivided into oral medications and topical medications. The disease-specific quality of life was assessed using the Chinese version of the 31-item rhinosinusitis outcome measure (CRSOM-31) [9]. The CRSOM-31 is a validated instrument translated from the widely used rhinosinusitis outcome measure (RSOM-31) [10]. It contains seven domains including nasal symptoms (6 items), eye symptoms (3 items), sleep (3 items), ear symptoms (5 items), general symptoms (7 items), practical problems (4 items), and emotional consequences (3 items) for evaluation of the rhinitis or rhinosinusitis-related impact on the quality of life. For each symptom, there are two response scales: magnitude (0 to 5) and importance (1 to 4). The CRSOM-31 symptom-impact score is the product of the magnitude and importance scores, with higher scores indicating worse disease-specific quality of life. Etiologies of rhinitis were ascertained through medical records.

### 2.3. Statistical Analysis

Continuous data were expressed as mean ± standard deviation (SD), and categorical data were expressed as frequencies and percentages. Univariate logistic regression analyses were conducted to assess the odds ratios and 95% confidence intervals for each of the treatment modalities with the independent variables, including sex, age, body mass index (BMI), marital status, educational level, alcohol use, smoking, regular exercise, general health status, CRSOM-31 symptom-impact scores, and four etiologies of rhinitis. Multivariate logistic regression analyses with backward stepwise selection method were used to evaluate the independent factors associated with the use of each of the treatment modalities. In all regression analyses, age was categorized into five groups. BMI was calculated as weight (in kilograms) divided by height (in meters) squared. Based on the definition from the Bureau of Health Promotion, Department of Health, Taiwan, the respondents were categorized as underweight (BMI < 18.5 kg/m^2^), normal weight (BMI 18.5–23.9 kg/m^2^), overweight (BMI 24.0–26.9 kg/m^2^), or obese (BMI ≥ 27.0 kg/m^2^). Educational levels were divided into elementary school or lower (grade 1 to grade 6) and high school or above. General health status of the patients was grouped into three levels (poor or fair, good, and very good or excellent). CRSOM-31 symptom-impact scores were divided into tertiles. All computations were performed using SPSS version 17.0 (SPSS, Inc., Chicago, IL, USA). Two-tailed *P* values < 0.05 were considered statistically significant.

## 3. Results

A total of 279 patients with acute or chronic rhinosinusitis, allergic or nonallergic rhinitis were successfully interviewed between July and September 2011. The mean age of the patients was 48.6 years and 58.4% were males. The basic characteristics of the study participants are summarized in Table 1. 

In terms of the use of treatment modalities, 85.7% of the patients had used pharmacology (77.1% had used oral medications and 65.9% had used topical medications), 38.4% had used surgical intervention, 34.4% had used TCM, and 34.1% had used nasal irrigation (Table 2). In addition, 19 (6.8%) patients had not used any treatment modalities, and 56 (20.1%), 63 (22.6%), 77 (27.6%), 36 (12.9%), and 28 (10.0%) patients had used 1, 2, 3, 4, and 5 treatment modalities, respectively. Regarding the severity of the rhinitis, since only 56 patients (20%) used only a single treatment modality, there are too few individuals to provide statistically meaningful comparisons between the five treatment modalities. Nonetheless, when the total number of modalities (0 to 5) used by the patients was compared, we found that the CRSOM-31 scores in patients who had used all five modalities (269.4 ± 130.3) significantly higher compared to those who had used fewer modalities. No significant differences in CRSOM-31 scores were observed among those patients who had used 4 (196.6 ± 107.1), 3 (173.7 ± 85.8), 2 (162.6 ± 93.3), 1 (148.3 ± 101.4), or no (125.7 ± 88.7) treatment modalities.

Table 2 also showed the results of univariate logistic regression analyses for each of the six treatment modalities with all the independent variables. Furthermore, six separate multivariate logistic regression analyses were conducted to assess the association between each of the treatment modalities and their independent and significant factors (Table 3). Male sex was significantly associated with the use of surgical intervention. Educational levels of elementary school or below were significantly associated with the use of pharmacology treatment modality and also with the use of subgroup of pharmacology treatment modality (oral or topical medications). Fair or poor general health status was significantly associated with the use of pharmacology treatment modality and especially with the use of topical medications, whereas very good or excellent general health status was inversely associated with the use of surgical intervention. Smoking was significantly associated with the use of nasal irrigation. Alcohol use was significantly associated with the pharmacology treatment modality. Worse disease-specific quality of life (CRSOM-31 symptom-impact score) was significantly associated with the use of pharmacology treatment modality, oral medications, and traditional Chinese medicine. In terms of the etiologies of rhinitis, allergic rhinitis was significantly associated with topical medications. Deviation of nasal septum was significantly associated with surgical intervention. Finally, chronic rhinosinusitis, both with or without nasal polyps, was significantly associated with the use of topical medications, surgical intervention, and the use of nasal irrigation. 

## 4. Discussion

Rhinitis and rhinosinusitis are common health problems that impact on the quality of life of their sufferers [11, 12]. They also impose a substantial burden on the healthcare resources [13]. The World Health Organization proposed a stepwise treatment for allergic rhinitis in the Allergic Rhinitis and its Impact on Asthma (ARIA) guideline [2]. Oral antihistamine and topical corticosteroid are recommended for the first-line treatment for mild and moderate-severe allergic rhinitis by the ARIA guideline. Our results showed that most rhinitis patients had received pharmacological treatments (85.7%) which included the uses of oral medications (77.1%) and topical medications (65.9%), indicating that guideline-directed rhinitis treatment is widely adopted by both physicians and patients in Taiwan. In addition, our data showed that approximately a third of the patients (34.1%) had used nasal irrigation to relieve their rhinitis symptoms. Nasal irrigation is used for various sinonasal condition and postoperative care after nasal surgery [14–16]. The mechanisms of nasal irrigation include physical cleaning, enhancement of mucociliary function, and removal of local inflammatory mediators. Although it is widely recommended and commonly used in Taiwanese patients, there was no data reporting the proportion of nasal irrigation use in clinical settings. 

About one third of our patients (34.4%) had used TCM for their rhinitis symptoms. The relatively common use of it could partly be explained by the health care system of Taiwan because TCM services are fully reimbursed by the National Health Insurance program [17]. Based on the results of an analysis of TCM outpatient reimbursement claims from 1996 to 2001 in Taiwan, diseases of the respiratory system were found to rank at the top of the major disease categories for TCM visits, which accounted for 27% of all TCM visits [18]. 

Understanding the factors associated with the use of different treatment modalities for rhinitis can be helpful for clinicians when they are considering the choice of treatment for their patients. Our results demonstrated that sex, educational levels, general health status, smoking habit, alcohol use, and disease-specific quality of life were independent factors associated with different treatment modalities. These associations had been adjusted for the etiologies of rhinitis and remained statistically significant. In particular, male patients were more likely to receive surgical interventions, while lower educational levels were associated with the use of oral and topical medications.

Patients with poor or fair general health status were associated with the use of topical medications. A possible reason is that the occurrence of drug-to-drug interactions is relatively rare with topical medications and therefore, suitable for patients who were on medications for their other diseases. It is of interest to note that very good or excellent general health status was inversely associated with surgical intervention. Since surgery for rhinitis is typically performed under local anesthesia, the general health status of a patient should not be a contraindication for surgery. The reasons for the association between the use of surgical intervention and general health status will require further investigations. 

Nasal irrigation is a method to relieve sinus symptoms by rinsing the nasal cavity with isotonic or hypertonic saline. A Cochrane systematic review on eight randomized controlled trials concluded that the use of topical saline could be included as a treatment adjunct for the symptoms of chronic rhinosinusitis [19]. In our study, smoking was significantly associated with the use of nasal irrigation. Nasal irrigation has been claimed as a method to remove nicotine and tar buildup in the nasal passages for smokers, and this might explain the association between nasal irrigation and smoking observed in this study. Previous large-scale population studies have revealed that cigarette smoking and chronic rhinitis were associated in a dose-dependent manner [20]. In addition, past and current secondhand tobacco smoke exposure was reported to be a risk factor for allergic rhinitis. Subjects with secondhand tobacco smoke exposure and allergic rhinitis were significantly more likely to use nasal decongestants [21]. However, no studies have specifically investigated whether smoking was associated with increased use of nasal irrigation. 

Finally, worse disease-specific quality of life, assessed by the use of CRSOM-31, was found to be associated with the use of oral medications and TCM. Since it is likely that the chronicity of rhinitis symptoms is associated with a worse quality of life, patients with worse quality of life might seek alternative therapies including TCM as a way to minimize the adverse effects of chronic use of conventional medications [22]. This might explain the association between CRSOM-31 and the use of TCM. The association between CRSOM-31 and the use of oral medications could be explained by the fact that oral medication is often used for moderate or severe cases not responsive to topical medications, which are likely to be cases with worse quality of life.

This study has some limitations that need to be considered. First, we did not directly measure the severity of rhinitis in our patients. It is possible that the choice of treatment modalities was affected by disease severity. Nevertheless, we have included CRSOM-31 symptom-impact scores in our multivariate analyses as a proxy to control for the effect of severity of rhinitis. Second, all patients were recruited from one medical center, and most of them had previously been treated for rhinitis by their primary care physicians. Therefore, they might represent cases of greater severity. 

## 5. Conclusions

Results from the present study indicated that common treatment modalities in Taiwanese patients with rhinitis included oral medications, topical medications, surgical intervention, TCM, and nasal irrigation. The use of different treatment modalities among Taiwanese patients with rhinitis was associated with different factors including sex, educational levels, general health status, smoking habit, alcohol use, and disease-specific quality of life, adjusting for etiologies of rhinitis.

## Figures and Tables

**Table 1:** Characteristics of study participants (*N* = 279).

Characteristic	*n* (%)
Sex	
Female	116 (41.6)
Male	163 (58.4)
Age (yr)	48.6 ± 18.1 (50.0, 20–89)*
≤30	54 (19.4)
31–40	46 (16.5)
41–50	47 (16.8)
51–60	69 (24.7)
>60	63 (22.6)
Body mass index (kg/m^2^)	24.0 ± 3.8 (23.8, 12.3–35.9)*
Underweight	12 (4.3)
Normal	141 (50.5)
Overweight	72 (25.8)
Obese	54 (19.4)
Marital status	
Single, divorced, widowed, or other	94 (33.7)
Married	185 (66.3)
Educational level	
Elementary school or below	78 (28.0)
High school or above	201 (72.0)
General health status	
Poor or fair	55 (19.7)
Good	158 (56.6)
Very good or excellent	66 (23.7)
Smoking	
No	232 (83.2)
Yes	47 (16.8)
Alcohol use	
No	208 (74.6)
Yes	71 (25.4)
Exercise	
No	52 (18.6)
Yes	227 (81.4)
CRSOM-31 symptom-impact score	175.4 ± 104.3 (158.0, 2–472)*
<116	94 (33.7)
117–205	92 (33.0)
>205	93 (33.3)
CRSOM-31 nasal symptoms score	42.0 ± 24.0 (39.0, 0–108)
CRSOM-31 eye symptoms score	9.1 ± 10.2 (5.0, 0–40)
CRSOM-31 sleep score	21.9 ± 18.2 (19.0, 0–60)
CRSOM-31 ear symptoms score	15.8 ± 16.2 (12.0, 0–66)
CRSOM-31 general symptoms score	35.5 ± 29.5 (28.0, 0–135)
CRSOM-31 practical problems score	29.4 ± 20.4 (25.0, 0–91)
CRSOM-31 emotional consequences score	21.8 ± 17.8 (20.0, 0–60)
Etiology of rhinitis	
Allergic rhinitis	
No	132 (47.3)
Yes	147 (52.7)
Deviation of nasal septum	
No	234 (83.9)
Yes	45 (16.1)
Chronic hypertrophic rhinitis	
No	194 (69.5)
Yes	85 (30.5)
Chronic rhinosinusitis	
No	127 (45.5)
Without nasal polyps	50 (17.9)
With nasal polyps	102 (36.6)

*Mean ± standard deviation (median, minimum–maximum).

**Table 2:** Univariate logistic regression analysis of factors associated with the use of treatment modalities in patients with rhinitis (*N* = 279).

Variable	Odds ratio (95% confidence interval), *P*					
	Pharmacology 239 (85.7%)	Subgroup of pharmacology		Surgical intervention 107 (38.4%)	Traditional Chinese medicine 96 (34.4%)	Nasal irrigation 95 (34.1%)
		Oral medications 215 (77.1%)	Topical medications 184 (65.9%)			
Sex						
Female	1.00	1.00	1.00	1.00	1.00	1.00
Male	0.82 (0.41–1.64), 0.572	0.87 (0.50–1.55), 0.642	0.91 (0.55–1.50), 0.701	1.61 (0.98–2.65), 0.062	1.06 (0.64–1.76), 0.815	1.35 (0.81–2.24), 0.250
Age (yr)						
≤30	1.00	1.00	1.00	1.00	1.00	1.00
31–40	2.34 (0.76–7.24), 0.139	2.06 (0.82–5.17), 0.126	1.57 (0.69–3.60), 0.286	1.11 (0.50–2.47), 0.806	1.16 (0.53–2.55), 0.712	3.44 (1.47–8.06), 0.004
41–50	1.39 (0.52–3.77), 0.514	1.31 (0.56–3.07), 0.538	1.47 (0.65–3.33), 0.359	0.98 (0.44–2.18), 0.951	0.79 (0.36–1.74), 0.553	2.14 (0.91–5.03), 0.081
51–60	1.51 (0.61–3.74), 0.377	1.96 (0.87–4.44), 0.104	1.29 (0.62–2.69), 0.498	1.62 (0.79, 3.33), 0.192	0.44 (0.21, 0.94), 0.033	1.68 (0.76–3.73), 0.201
>60	4.22 (1.27–13.98), 0.019	2.65 (1.10, 6.40), 0.030	1.48 (0.69, 3.16), 0.313	0.45 (0.20, 1.01), 0.052	0.22 (0.09–0.52), 0.001	0.99 (0.42–2.31), 0.973
Body mass index (kg/m^2^)						
Normal	1.00	1.00	1.00	1.00	1.00	1.00
Underweight	1.19 (0.48–2.92), 0.707	0.87 (0.41–1.83), 0.704	1.25 (0.66–2.38), 0.495	1.28 (0.66–2.47), 0.464	1.24 (0.64–2.40), 0.521	0.85 (0.44–1.64), 0.626
Overweight	1.91 (0.22–16.93), 0.560	3.14 (0.37–26.86), 0.295	1.38 (0.37–5.13), 0.636	0.67 (0.16–2.77), 0.577	0.67 (0.16–2.77), 0.577	0.34 (0.07–1.71), 0.191
Obese	0.79 (0.30–2.06), 0.630	1.00 (0.43–2.34), >0.999	1.92 (0.90–4.08), 0.091	1.51 (0.73–3.15), 0.269	0.82 (0.39–1.76), 0.617	0.96 (0.46–2.00), 0.915
Marital status						
Single, divorced, widowed, or other	1.00	1.00	1.00	1.00	1.00	1.00
Married	0.82 (0.40–1.70), 0.594	0.95 (0.53–1.72), 0.865	1.15 (0.68–1.94), 0.594	0.82 (0.49–1.36), 0.443	0.55 (0.33–0.92), 0.022	0.93 (0.55–1.57), 0.791
Educational level						
Elementary school or below	1.00	1.00	1.00	1.00	1.00	1.00
High school or above	0.25 (0.09–0.72), 0.011	0.46 (0.23–0.93), 0.031	0.44 (0.24–0.81), 0.008	1.07 (0.63–1.84), 0.802	1.93 (1.07–3.48), 0.029	0.89 (0.52–1.55), 0.685
General health status						
Good	1.00	1.00	1.00	1.00	1.00	1.00
Poor or fair	4.98 (1.14–21.78), 0.033	1.33 (0.61–2.90), 0.476	3.98 (1.69–9.37), 0.002	0.82 (0.44–1.53), 0.525	1.73 (0.93–3.22), 0.086	1.62 (0.87–3.05), 0.130
Very good or excellent	0.77 (0.37–1.61), 0.482	0.79 (0.41–1.52), 0.475	0.70 (0.39–1.25), 0.223	0.33 (0.17–0.64), 0.001	0.62 (0.32–1.18), 0.146	0.91 (0.49–1.70), 0.772
Smoking						
No	1.00	1.00	1.00	1.00	1.00	1.00
Yes	1.98 (0.67–5.84), 0.219	2.87 (1.08–7.58), 0.034	1.26 (0.64–2.50), 0.499	1.37 (0.73–2.58), 0.329	1.37 (0.72–2.60), 0.342	3.26 (1.71–6.20), <0.001
Alcohol use						
No	1.00	1.00	1.00	1.00	1.00	1.00
Yes	1.72 (0.73–4.09), 0.217	1.64 (0.82–3.29), 0.164	0.67 (0.39–1.18), 0.163	1.06 (0.61–1.85), 0.828	1.58 (0.91–2.74), 0.108	1.61 (0.92–2.80), 0.093
Exercise						
No	1.00	1.00	1.00	1.00	1.00	1.00
Yes	1.11 (0.48–2.57), 0.811	1.15 (0.57–2.32), 0.695	1.03 (0.55–1.94), 0.924	0.61 (0.33–1.12), 0.112	1.37 (0.71–2.64), 0.350	0.72 (0.38–1.33), 0.287
CRSOM-31 symptom-impact score						
<116	1.00	1.00	1.00	1.00	1.00	1.00
117–205	3.94 (1.60–9.70), 0.003	2.65 (1.32–5.33), 0.006	2.14 (1.16–3.94), 0.014	0.85 (0.46–1.57), 0.604	1.76 (0.89–3.47), 0.106	1.27 (0.68–2.37), 0.462
>205	2.69 (1.20–6.03), 0.016	2.31 (1.17–4.54), 0.016	2.17 (1.18–3.99), 0.012	1.73 (0.96–3.12), 0.066	5.13 (2.66–9.88), <0.001	1.89 (1.03–3.48), 0.041
Etiology of rhinitis						
Allergic rhinitis						
No	1.00	1.00	1.00	1.00	1.00	1.00
Yes	1.01 (0.52–1.97), 0.979	0.83 (0.47–1.46), 0.516	1.14 (0.70–1.87), 0.603	0.64 (0.39–1.04), 0.070	1.10 (0.67–1.80), 0.720	0.68 (0.41–1.12), 0.126
Deviation of nasal septum						
No	1.00	1.00	1.00	1.00	1.00	1.00
Yes	0.73 (0.31, 1.72), 0.473	0.91 (0.43–1.91), 0.793	0.92 (0.48–1.80), 0.816	0.87 (0.45–1.69), 0.674	2.07 (1.08–3.95), 0.028	0.85 (0.43–1.70), 0.650
Chronic hypertrophic rhinitis						
No	1.00	1.00	1.00	1.00	1.00	1.00
Yes	0.90 (0.44–1.84), 0.763	0.79 (0.44–1.43), 0.439	0.92 (0.54–1.58), 0.772	0.66 (0.39–1.14), 0.135	1.42 (0.84–2.41), 0.194	0.58 (0.33–1.02), 0.058
Chronic rhinosinusitis						
No	1.00	1.00	1.00	1.00	1.00	1.00
Without nasal polyps	1.66 (0.78–3.52), 0.188	1.80 (0.96–3.37), 0.068	1.64 (0.95–2.84), 0.078	2.37 (1.34–4.19), 0.003	0.94 (0.54–1.63), 0.810	2.10 (1.18–3.75), 0.012
With nasal polyps	1.99 (0.71, 5.57), 0.190	2.16 (0.93–5.04), 0.075	2.54 (1.19–5.41), 0.016	8.69 (4.13–18.28), <0.001	1.30 (0.67–2.56), 0.443	4.88 (2.42–9.84), <0.001

**Table 3:** Multivariate logistic regression analysis of factors associated with the use of treatment modalities in patients with rhinitis (*N* = 279).

Variable	Odds ratio (95% confidence interval), *P*					
	pharmacology 239 (85.7%)	Subgroup of Pharmacology		Surgical intervention 107 (38.4%)	Traditional Chinese medicine 96 (34.4%)	Nasal irrigation 95 (34.1%)
		Oral medications 215 (77.1%)	Topical medications 184 (65.9%)			
Sex						
Female				1.00		
Male				2.11 (1.20–3.70), 0.009		
Educational level High school or above Elementary school or below	1.00 5.23 (1.74–15.73), 0.003	1.00 2.31 (1.12–4.76), 0.024	1.00 2.50 (1.32–4.74), 0.005			
General health status						
Good	1.00		1.00	1.00		
Poor or fair	6.48 (1.41–29.86), 0.016		4.47 (1.85–10.80), 0.001	0.92 (0.47–1.81), 0.805		
Very good or excellent	1.15 (0.51–2.60), 0.746		0.78 (0.42–1.42), 0.409	0.32 (0.15–0.66), 0.002		
Smoking						
No						1.00
Yes						2.72 (1.39–5.30), 0.003
Alcohol use						
No	1.00					
Yes	2.52 (1.01–6.27), 0.047					
CRSOM-31 symptom-impact score <116 117–205 >205	1.00 4.35 (1.68–11.28), 0.002 2.20 (0.91–5.33), 0.081	1.00 2.70 (1.33–5.46), 0.006 2.46 (1.24–4.89), 0.010			1.00 1.79 (0.90–3.57), 0.098 5.43 (2.79–10.60), <0.001	
Allergic rhinitis No Yes			1.00 2.37 (1.16, 4.86), 0.018			
Deviation of nasal septum No Yes					1.00 2.37 (1.19–4.72), 0.014	
Chronic rhinosinusitis No Without nasal polyps With nasal polyps			1.00 2.55 (1.18–5.51), 0.018 3.76 (1.54–9.13), 0.004	1.00 2.11 (1.20–3.70), 0.009 2.45 (1.35–4.44), 0.003		1.00 1.93 (1.07–3.49), 0.029 4.28 (2.09–8.76), <0.001

Other variables evaluated during model development included age, body mass index, marital status, exercise, and chronic hypertrophic rhinitis.

## List of Abbreviations

CRSOM-31: Chinese version of the 31-item rhinosinusitis outcome measure
TCM: Traditional Chinese medicine.
